# *Notes from the Field:* COVID-19–Associated Mucormycosis — Arkansas, July–September 2021

**DOI:** 10.15585/mmwr.mm7050a3

**Published:** 2021-12-17

**Authors:** Theresa M. Dulski, Megan DeLong, Kelley Garner, Naveen Patil, Michael J. Cima, Laura Rothfeldt, Trent Gulley, Austin Porter, Keyur S. Vyas, Hazel K. Liverett, Mitsuru Toda, Jeremy A.W. Gold, Atul Kothari

**Affiliations:** ^1^Epidemic Intelligence Service, CDC; ^2^Arkansas Department of Health; ^3^University of Arkansas for Medical Sciences, Little Rock, Arkansas; ^4^Mycotic Diseases Branch, Division of Foodborne, Waterborne, and Environmental Diseases, National Center for Emerging and Zoonotic Infectious Diseases, CDC.

During September 17–24, 2021, three clinicians independently notified the Arkansas Department of Health (ADH) of multiple patients with mucormycosis after a recent diagnosis of COVID-19. To provide data to guide clinical and public health practice, ADH coordinated a statewide call on October 11, 2021 to infection preventionists for COVID-19–associated mucormycosis cases.

Mucormycosis is an uncommon but severe invasive fungal infection caused by molds in the order Mucorales. Mucormycosis typically affects persons with immunocompromising conditions such as a hematologic malignancy, stem cell or solid organ transplantation, or uncontrolled diabetes (*1*). The emergence of COVID-19–associated mucormycosis has been described in other parts of the world, particularly in India, but has been infrequently reported in the United States (*2*–*4*). COVID-19 might increase mucormycosis risk because of COVID-19–induced immune dysregulation or associated treatments such as corticosteroids and immunomodulatory drugs (e.g., tocilizumab or baricitinib) that impair host defenses against molds (*5*).

A case of mucormycosis was defined as laboratory identification of Mucorales by culture, histopathology, or polymerase chain reaction in a patient with a clinical diagnosis of invasive mucormycosis.^†^ Cases were considered COVID-19–associated if the patient received a positive reverse transcription–polymerase chain reaction or antigen test result for SARS-CoV-2 (the virus that causes COVID-19) during the 60 days preceding the mucormycosis diagnosis. Cases were reported to ADH using a standardized case report form, medical records, or oral report. Data were stored using Research Electronic Data Capture software (version 10.6.18; Vanderbilt University) (*6*) and linked to state vital records and state immunization and COVID-19 registries. Patient demographic characteristics, underlying conditions, clinical course, treatment, and clinical outcomes were examined. This activity was reviewed by CDC and was conducted consistent with applicable federal law and CDC policy.^§^

Ten COVID-19–associated mucormycosis cases that occurred during July 12–September 28, 2021, were reported to ADH by six hospitals.^¶^ Nine patients lived in Arkansas, with patients representing each of the state’s five public health unit regions; one patient lived in a bordering state. Among all 10 patients, the median age was 57 years (range = 17–78 years), all patients were non-Hispanic White persons, seven were male, one had a history of solid organ transplantation, and one had a history of recent traumatic injury at the body site where mucormycosis later developed. Eight patients had diabetes; among these, the median hemoglobin A1c was 8.6% (range = 6.0%–14.3% [normal <5.7%]).** During hospitalization, three patients with diabetes experienced diabetic ketoacidosis. Mucormycosis clinical signs and symptoms included those that were rhino-orbital (four patients, including three with cerebral involvement), pulmonary (three), disseminated (two), and gastrointestinal (one).

The median interval from COVID-19 diagnosis to the first positive test result for mucormycosis was 18.5 days (range = 6–52 days). None of the patients had been vaccinated against COVID-19. COVID-19 treatment included supplemental oxygen therapy (eight patients), invasive mechanical ventilation (five), corticosteroids (nine), tocilizumab (two), and baricitinib (two). Five patients received surgical treatment to excise mucormycosis-affected tissue. Six of the 10 patients died during hospitalization or within 1 week of discharge.

The findings in this report are subject to at least two limitations. First, cases were identified using passive reporting, which could have missed some mucormycosis cases. Second, the definition of COVID-19–associated cases was limited to positive tests within 60 days preceding mucormycosis diagnosis, which could have missed some cases occurring outside this period.

The 10 reported COVID-19–associated mucormycosis cases occurred during a 79-day period (July 12–September 28, 2021) coinciding with a statewide surge in COVID-19 cases caused by the highly transmissible SARS-CoV-2 B.1.617.2 (Delta) variant.^††^ By comparison, nine mucormycosis cases per year might be expected in Arkansas (population approximately 3,000,000)^§§^ based on the estimated U.S. incidence of mucormycosis hospitalizations (approximately three per 1,000,000 persons annually) (*7*). The reported COVID-19–associated mucormycosis cases might have occurred because of COVID-19–induced immune dysregulation or medical treatments (*5*).

Because of the severity of mucormycosis, it is important that clinicians maintain a high index of suspicion for COVID-19–associated mucormycosis, including in patients without severe immunocompromising conditions. Mucormycosis treatment guidelines recommend prompt antifungal therapy^¶¶^ and surgical intervention to improve outcomes (*8*). Maintenance of glycemic control in patients with diabetes, guideline-based use of corticosteroids for COVID-19 treatment,*** and vaccination against COVID-19 should be encouraged. As a result of these reported cases, ADH sent an update on the statewide Health Alert Network (October 21, 2021) and nationwide Epi-X listserv (October 22, 2021) to improve mucormycosis prevention, diagnosis, and treatment. COVID-19–associated mucormycosis surveillance and case investigations are ongoing.

## Footnotes

† Signs and symptoms of mucormycosis vary by the affected body site. Rhino-orbital-cerebral mucormycosis signs and symptoms frequently include fever, unilateral facial swelling, headache, sinus congestion, vision loss, proptosis, and necrotic lesions of the nasal bridge or palate. Cutaneous mucormycosis signs and symptoms frequently include fever, blisters or ulcers that become necrotic, and pain, erythema, or swelling around the wound. Pulmonary mucormycosis signs and symptoms frequently include fever, cough, chest pain, and shortness of breath. Gastrointestinal mucormycosis signs and symptoms frequently include fever, abdominal pain, nausea, vomiting, and gastrointestinal bleeding. https://www.cdc.gov/fungal/diseases/mucormycosis/symptoms.html

§ 45 C.F.R. part 46.102(l)(2), 21 C.F.R. part 56; 42 U.S.C. Sect. 241(d); 5 U.S.C. Sect. 552a; 44 U.S.C. Sect. 3501 et seq.

¶ Three additional potential cases were reported to ADH but were excluded from this report, two because the patients lacked clinical evidence of invasive mucormycosis and the other because the interval from COVID-19 diagnosis and mucormycosis diagnosis exceeded 60 days.

** One patient did not have a recent hemoglobin A1c result available. https://www.cdc.gov/diabetes/managing/managing-blood-sugar/a1c.html

††
https://www.healthy.arkansas.gov/programs-services/topics/novel-coronavirus

§§
https://www.census.gov/quickfacts/AR

¶¶ Antifungal drugs that are effective against mucormycosis include amphotericin B, posaconazole, and isavuconazole. Other antifungal drugs, including fluconazole, voriconazole, and echinocandins, are not effective for treating mucormycosis. https://www.cdc.gov/fungal/diseases/mucormycosis/treatment.html

***
https://www.covid19treatmentguidelines.nih.gov/management/clinical-management/hospitalized-adults--therapeutic-management/
